# Enhancement of Digestive Enzyme Activity in *Enterococcus faecalis* Using ARTP Mutagenesis

**DOI:** 10.3390/microorganisms12122425

**Published:** 2024-11-25

**Authors:** Meng Yuan, Zhengzhong Li, Qunlan Zhou, Xiaochuan Zheng, Cunxin Sun, Bo Liu, Aimin Wang, Aimin Zhu

**Affiliations:** 1 College of Fisheries and Life Science, Shanghai Ocean University, Shanghai 201306, China; m220150563@st.shou.edu.cn; 2 Key Laboratory of Freshwater Fisheries and Germplasm Resources Utilization, Ministry of Agriculture and Rural Affairs, Freshwater Fisheries Research Center, Chinese Academy of Fishery Sciences, Wuxi 214081, China; ymlizz@163.com (Z.L.); zhouql@ffrc.cn (Q.Z.); zhengxiaochuan@ffrc.cn (X.Z.); 3 Wuxi Fisheries College, Nanjing Agricultural University, Wuxi 214128, China; 4 College of Marine and Biology Engineering, Yancheng Institute of Technology, Yancheng 224051, China; blueseawam@ycit.cn; 5 Yancheng Academy of Fishery Science, Yancheng 224051, China; zam--3@163.com

**Keywords:** atmospheric and room temperature plasma, enterococcus faecalis, α-amylase, protease, lipase, antioxidant capacity

## Abstract

*Enterococcus faecalis* is used as a probiotic in animal and human food supplements. Atmospheric and room temperature plasma (ARTP) systems have frequently been used to screen for effective mutant probiotics. In this study, *E. faecalis* was treated with ARTP, and high-yielding digestive enzyme mutant strains were obtained by measuring the activities of α-amylase, lipase, and neutral protease. A total of 833 mutant strains were obtained after 40–60 s of ARTP treatment, and after screening for digestive enzyme activity, EF-448, EF-798, and EF-804 were obtained. The three strains demonstrated an 180% increase in α-amylase activity, a 30% increase in lipase activity, and a more than 40% increase in neutral protease activity. Furthermore, the enzyme activities remained stable after nine generations. In addition, the strains exhibited high auto-aggregation capacity (over 91%) and high cell hydrophobicity (over 93%). After exposure to simulated intestinal fluid for 6 h, the survival rates of EF-448 and EF-798 were 85.71% and 82.32%, respectively. Moreover, the three mutant strains retained antioxidant capacity and DPPH free radical scavenging ability, and there was no hemolysis. A safety experiment has shown that there is no mortality of *Macrobrachium rosenbergii* within 14 days after receiving injections of mutant strains at different concentrations. In conclusion, this study obtained three mutant strains with high production of digestive enzymes and stable inheritance through ARTP mutagenesis of *E. faecalis*, providing an efficient microbial resource.

## 1. Introduction

In recent years, numerous studies have shown that probiotics are beneficial to humans and animals as food products or feed additives, including *Lactobacillus plantarum*, *Bifidobacterium*, *Bacillus subtilis*, and others [1,2]. For aquatic animals, probiotics as feed additives can promote growth, increase immunity, and improve the gut microbiota environment [3]. *Enterococcus faecalis*, as a dominant bacterial strain in human and animal intestines, naturally colonizes and plays an important role in maintaining the homeostasis of the intestinal environment. It has been proven to participate in the digestion, absorption, and metabolism of nutrients. Owing to its potent probiotic characteristics, *E. faecalis* has garnered significant interest in the feed sector [4]. Unlike mammals, crustaceans possess short intestines and lack robust mechanical digestive capabilities. Consequently, their digestion mostly depends on the organism’s secretion of digestive enzymes [5]. The application of exogenous enzymes in aquaculture can improve growth performance and boost the health of aquatic organisms [6]. Furthermore, the use of digestive enzyme supplements in the baseline diet of animals might improve feed consumption and promote their growth performance [7,8].

Atmospheric and Room Temperature Plasma (ARTP) is a whole-cell mutagenesis tool based on high-frequency atmospheric pressure luminescent discharge plasma [8,9]. The principle of mutagenesis of ARTP, briefly, can produce a variety of mutant strains by generating active high-energy particles capable of altering a cell’s structure and permeability, leading to DNA damage and forcing the cell to activate the save our souls (SOS) repair mechanism [8,10]. These active agents attack the O-H, O-P, and N-C glycosidic bonds in the structure of the DNA chain, thereby disrupting the molecular structure of the DNA and leading to single-stranded DNA breaks. Double-stranded DNA breaks occur when the breakpoints in different single-stranded regions are in close proximity with each other [11]. Currently, ARTP mutagenesis has been used in 24 bacterial and 14 fungus species successfully [9]. Studies have demonstrated that ARTP can affect the genetic material and protein structure of micro-organisms. It could increase the chitosan production capacity of *Bacillus* [12,13], the surfactant production of *Bacillus subtilis* [14], the acid tolerance of *Lactobacillus acidophilus* [15], the antimicrobial capacity of *Brevibacillus* sp. SPR19, and the tolerance and adsorption capacity of *Bacillus velezensis* for hexavalent chromium [16,17].

The effects of ARTP on the characteristics, genetic stability analysis, and safety evaluation of *E. faecalis* for high digestive enzyme activities in aquatic animals remain poorly understood. Therefore, in this study, ARTP was used to mutate *E. faecalis* to screen for strains with enhanced capabilities for producing digestive enzymes, including α-amylase, protease, and lipase. Furthermore, the safety, genetic stability, and tolerance of the mutant strains also will be investigated. This will provide a theoretical basis and stable and efficient strain resources for further in-depth research in the feed probiotics industry.

## 2. Materials and Methods

### 2.1. Strain and Culture Conditions

*E. faecalis* was provided by the Freshwater Fisheries Research Center of Chinese Academy of Fishery Sciences (Wuxi, China). The strain was stored in 50% glycerol at −80 °C. *E. faecalis* was cultured at 37 °C for 24 h in De Man, Rogosa Sharpe (MRS) (Qingdao Hi-tech Industrial Park Hope Bio-technology Co., Ltd., Qingdao, China) solid medium. A single colony was transferred to MRS broth and cultivated at 180 rpm and 37 °C for 24 h. All media were sterilized at 121 °C for 20 min [18].

### 2.2. Growth Curve

*E. faecalis* was inoculated (1%, *v*/*v*) in MRS broth and cultivated at 180 rpm and 37 °C for 24 h. A total volume of 200 μL of sample was absorbed to determine the value of OD600 at 0, 3, 6, 9, 12, 15, 18, 21, and 24 h [18]. The curve was plotted with time on the *X*-axis and OD600 on the *Y*-axis. The end of the logarithmic growth phase was taken as the optimum age for ARTP mutagenesis. Three replicates were performed.

### 2.3. Mutagenic Bacteria Suspension Preparation

*E. faecalis* was cultured in MRS solid medium at 37 °C for 24 h. Single colonies were transferred to MRS broth and cultivated at 180 rpm and 37 °C for 18 h. After dilution to 1.0 × 10^7^ CFU/mL with sterile saline, 1.5 mL bacteria solution was taken and centrifuged at 5000 r/min for 10 min at 4 °C, and the pellet was washed three times with sterile saline and finally suspended in saline. We added 5% glycerol for moisturizing.

### 2.4. Mutagenesis by ARTP

The ARTP mutagenesis experiment was conducted according to the procedures outlined by Ma et al. [19]. Specifically, 10 μL of the bacterial suspension was transferred onto a sterile slide and then subjected to mutagenesis using an ARTP mutagenic apparatus (Wuxi Yuanqing Tianmu Biological Technology Co., Ltd., Wuxi, China). RF power was set to 100 W, the helium gas flow rate was 10 SLM, and the mutagenesis time was set to 0, 10, 20, 30, 40, 50, 60, 80, 100, and 120 s. The treated cells were serially diluted to the requisite concentration and inoculated onto MRS agar in order to assess cell viability. Lethality rate was calculated in accordance with the following formula:

Lethality rate = (A − B)/A × 100%(1)

Notes: A represents the colony number of the control group, while B represents the colony number of the mutant group.

### 2.5. Pre-Screening and Re-Screening

#### 2.5.1. Pre-Screening

The optimal irradiation time was selected according to the growth curve. A single colony was picked for activation after mutagenesis. After strain rejuvenation, single colonies of *E. faecalis* and its mutant strains were transferred to MRS broth, cultivated at 180 rpm and 37 °C for 18 h, and then centrifuged at 5000 r/min for 10 min. The α-amylase activity was quantified by the kit of Nanjing Jiancheng Bioengineering Institute (Nanjing, China. Kit code: C016-1-1). α-Amylase measurement principle: α-amylase hydrolyses starch to form glucose, maltose, and dextrin. If the substrate concentration is known and in excess, iodine solution is added to bind to the unhydrolyzed starch to form a blue complex, and the amount of starch hydrolyzed can be calculated from the shade of blue to calculate amylase activity. Three replicates were performed.

#### 2.5.2. Re-Screening

The best 10% of the higher α-amylase yields was used to prepare the supernatant of the fermentation broth according to Section 2.5.1 for lipase and neutral protease activity. Lipase activities were quantified by the commercial detection kits of Beijing Solarbio Science and Technology Co., Ltd. (Beijing, China. Kit code: BC2345). Neutral protease activity was quantified by the commercial detection kits of Suzhou Keming Biotechnology Co., Ltd. (Suzhou, China, Kit code: NPT-2-W). Lipase measurement principle: lipase catalyzed the hydrolysis of oil esters into fatty acids. The formation rate of fatty acids was determined by the copper soap method. Neutral proteases measurement principle: neutral proteases catalyst the hydrolysis of casein to produce tyrosine. Three replicates were performed.

#### 2.5.3. Overall Rating

The overall rating was calculated according to the formula:

Overall rating (%) = (α-amylase activity/maximum α-amylase activity) × 100 × 0.4 + (neutral protease activity/maximum neutral protease activity) × 100 × 0.4 + (lipase activity/maximum lipase activity) × 100 × 0.2(2)

### 2.6. The Genetic Stability Analysis of the Mutant

The genetic stability experiment was performed according to Xu et al. [14]. *E. faecalis* was cultured in MRS solid medium at 37 °C for 24 h. Single colonies were transferred to a new MRS solid medium and cultivated at 37 °C for 24 h. Nine consecutive generations were inoculated, and the activities of digestive enzymes (α-amylase, neutral protease, and lipase) were determined at generation 1, 3, 6, and 9. Experiments were performed in triplicate.

### 2.7. Evaluation of Probiotic Characteristics

#### 2.7.1. Auto-Aggregation of Selected Mutant Isolates

Auto-aggregation was carried out according to Angmo et al. with little modification [20]. *E. faecalis* and the three mutant isolates screened were inoculated in MRS broth at 180 rpm and 37 °C for 18 h. The cell suspensions were centrifuged at 4000 rpm for 10 min. The washed pellets were resuspended in PBS (0.1 M, pH 7.2). The OD_600_ of the bacterial suspension was adjusted to 1.0 ± 0.05. The absorbance was measured at 600 nm. Experiments were performed in triplicate. Samples were monitored with different time intervals (0, 2, 4, and 24 h), and the auto-aggregation percentage was calculated as follows:

Auto-aggregation (%) = (1 − A_t_/A_0_) × 100 (3)

Note: A_t_ denotes the absorbance at time t h, while A_0_ is that at time 0 h.

#### 2.7.2. Cell Surface Hydrophobicity of Selected Mutant Isolates

The hydrophobicity test was performed according to the methods described by Jin et al. [21]. Bacterial suspensions are prepared in the same way as in Section 2.7.1. The xylene (2 mL) and bacterial suspension (2 mL) were mixed with equal volumes, vortexed thoroughly for 3 min, and let to stand at 37 °C for 40 min to stratify. The aqueous phase was then separated with a pipette and the absorbance was measured at OD_600_. Experiments were performed in triplicate. The hydrophobicity of xylene was calculated as follows:

Hydrophobicity (%) = (1 − A/A_0_) × 100% (4)

Notes: A_0_ and A are the absorbance (at OD_600_) before and after the treatment.

#### 2.7.3. Tolerance Analysis of Screened Strains

The intestinal juice tolerance assessment was performed according to Shi et al. with little modification [22]. *E. faecalis* and the three mutant isolates screened were inoculated in an MRS liquid medium at 180 r/min and 37 °C for 18 h. The OD*_600_* of the bacterial suspension was adjusted to 1.0 ± 0.05 with sterile PBS. The suspension was inoculated into a test tube containing 5 mL of simulated intestinal fluid at an inoculum volume of 10%, shaken and mixed well, and then placed in a static water bath at 37 °C for 6 h. Samples were taken at 0, 2, 4, and 6 h of incubation, and the number of viable bacteria and the survival rate were determined. Three replicates per strain were prepared in parallel.

Survival rate (%) = (A_t_/A_0_) × 100% (5)

Notes: A_t_ is the number of surviving colonies after 0, 2, 4, and 6 h of incubation, and A_0_ is the number of surviving colonies at 0 h.

#### 2.7.4. Determination of the Antioxidant Capacity of the Mutant Strains

An assay kit (Jiancheng Bioengineering Institute, Nanjing, China. Kit code: A015-3-1) was used to measure the level of total antioxidant capacity (T-AOC) in fermentation supernatants, following the instructions provided by the manufacturer. The measurement technique involves the reduction of Fe^3+^-TPTZ to create blue Fe^2+^-TPTZ by antioxidants under acidic circumstances. T-AOC of the sample was determined by measuring the absorbance at 593 nm.

The 2,2-diphenyl-1-picrylhydrazyl (DPPH) free radicals of samples were determined by an assay kit (Jiancheng Bioengineering Institute, Nanjing, China. Kit code: A153-1-1). The measurement basis of this approach relies on the characteristics of the DPPH radical, which include having a single electron, exhibiting significant absorption at 517 nm, and causing a purple color in its alcoholic solution.

### 2.8. Safety Evaluation

#### 2.8.1. Hemolytic Activity

The three strains were cultured at 37 °C for 18 h, and single colonies were picked and inoculated with a sterile inoculating ring on sterile Columbia blood solid medium (Qingdao Hi-tech Industrial Park Hope Bio-technology Co., Ltd., Qingdao, China) by the scribing method, which was set as the experimental group; *Aeromonas hydrophila* were cultured as the control group in a 28 °C incubator for 48 h, it was observed whether there was any hemolysis phenomenon around the strains, and photos were taken for the record.

#### 2.8.2. Mutagenic Strains Safety Testing

The safety evaluation was evaluated referring to the methods described by Divisekera et al. with little modification [23]. A total of 210 *Macrobrachium rosenbergii* (3.5 ± 0.05 g) were acclimatized in the control environment for 7 days and were randomly divided into 7 groups. The experiment was performed in triplicate. All groups were injected with 100 μL of EF-448, EF-798, and EF-804 at the concentration of 1.0 × 10^8^ and 1.0 × 10^10^ CFU/mL, respectively, and the control group was injected with 100 μL of sterile saline. Disease incidence and death were continuously monitored and recorded for 14 days. Autopsy was checked at the end of the experiment for signs of poisoning.

### 2.9. Statistics Analysis

The results are represented as the mean ± SEM. Statistical analysis of the data was performed using a two-tailed unpaired Student’s *t*-test with SPSS 20.0 software, and *p* < 0.05 was considered as a significantly difference.

## 3. Results

### 3.1. Growth Curve of E. faecalis

**Figure 1:**
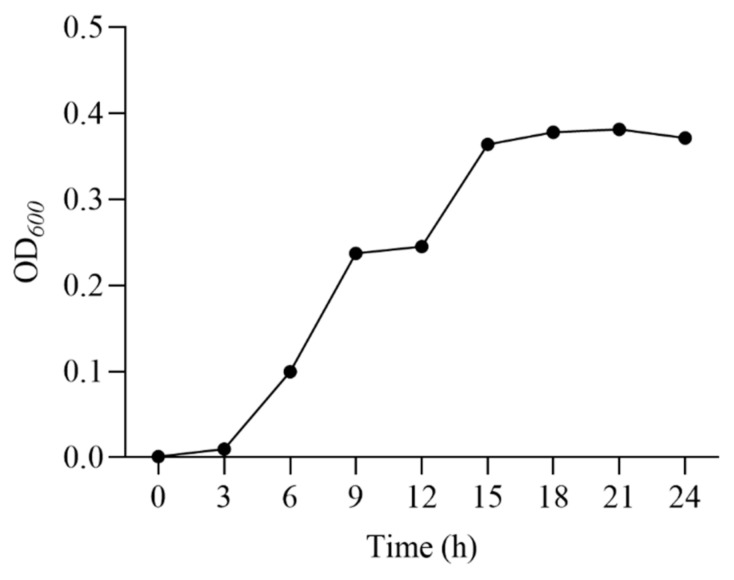
Growth curve of *E. faecalis*.

The growth curve of *E. faecalis* (Figure 1) showed that the strain grew rapidly from 0 to 18 h, and reached the decline phase at 24 h. Therefore, 18 h was chosen as the fermentation time for the seed solution.

### 3.2. ARTP Mutagenic Lethality Rate

**Figure 2:**
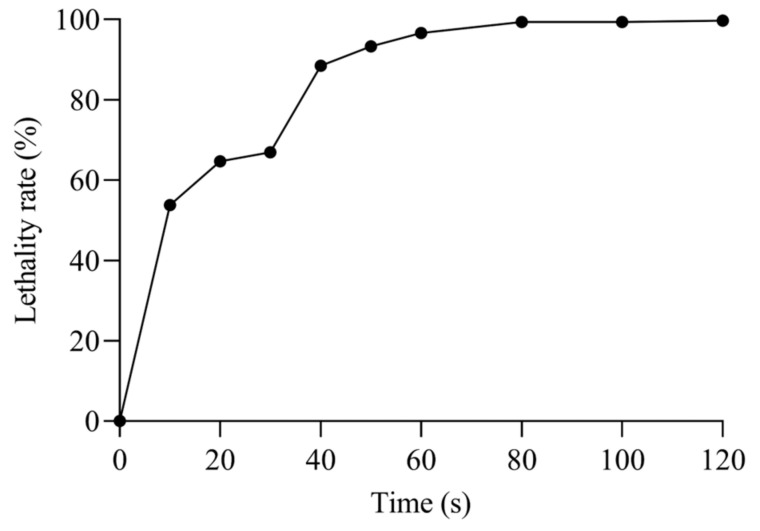
Effect of ARTP treatment time on the lethality rate of *E. faecalis*.

The lethality of *E. faecalis* increased proportionally with the duration of mutagenesis (Figure 2), with the mortality of the strain reaching 53.78% after 10 s of ARTP treatment and reaching 99% when the mutagenesis time exceeded 80 s, essentially killing all cells. When the lethality was 85–95%, the strain was prone to mutation, so the optimal mutagenicity times selected in this study were 40 s, 50 s, and 60 s.

### 3.3. Pre-Screening

**Figure 3:**
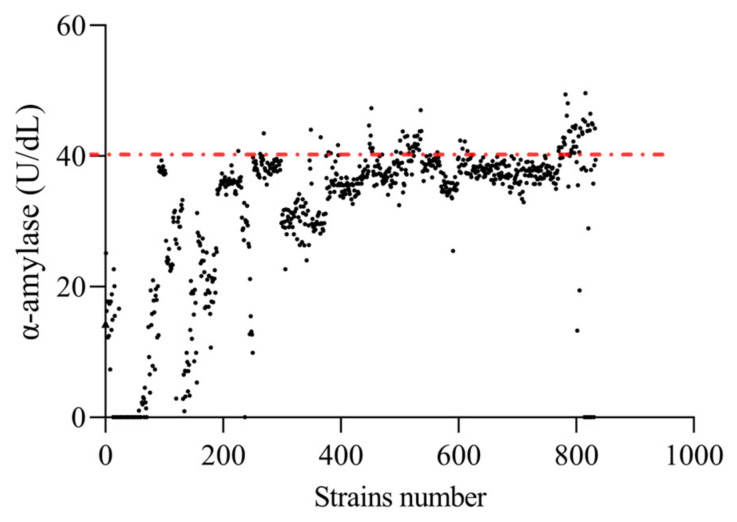
α-amylase activity of mutant strains. Black dots are the α-amylase activity producing activity of each strain and the red line is the boundary for the top 10% of strains.

A total of 833 single colonies (Figure 3), named EF-1 to EF-833, were selected. Among them, 698 strains showed a significant increase in α-amylase activity compared to the original strain (*p* < 0.05). Consequently, the top 10% of these strains were chosen for subsequent screening in this experiment, which amounted to 84 strains displaying an increase in α-amylase activity over 160% compared to that of the original strain.

### 3.4. Re-Screening

Lipase and neutral protease activities were determined by re-screening, and the combined score was calculated by combining with α-amylase (Table 1). There were 72 mutant strains with higher digestive enzyme combined scores than the original strain, and the six strains (EF-513, EF-804, EF-494, EF-798, EF-783, and EF-448) with the higher content of digestive enzymes were selected for the genetic stability experiment compared to the original strain of *E. faecalis* (EF, *p* < 0.05).

### 3.5. The Genetic Stability

**Figure 4:**
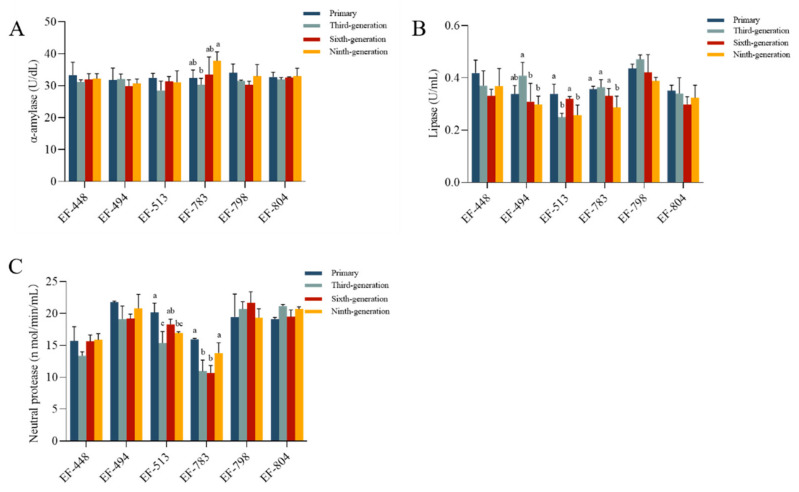
(**A**) α-amylase, (**B**) lipase, and (**C**) neutral protease of genetic stability in the mutant strains. Bars with different letters for each mean value indicate statistically significant differences (*p* < 0.05).

There was a significant difference in the α-amylase activity of EF-783 and the lipase activity of EF-494, EF-513, and EF-783 from primary to ninth generation (*p* < 0.05, Figure 4A,B). The neutral protease activities of EF-513 and EF-783 were significantly different from primary to ninth generation (*p* < 0.05, Figure 4C). The activities of α-amylase, lipase, and neutral protease of EF-448, EF-798, and EF-804 were not significantly different and were consistently maintained (*p* > 0.05). Consequently, the strains EF-448, EF-798, and EF-804 were utilized to assess their performance.

### 3.6. Hydrophobicity and Auto-Aggregation Ability of Mutant Strains

#### 3.6.1. Auto-Aggregation

Auto-aggregation rates of *E. faecalis*, EF-448, EF-798, and EF-804 showed similar trends at the different time points (2, 4, and 24 h), The data exhibited a progressive pattern and did not demonstrate a statistically significant distinction from that of different *E. faecalis* strains (*p* > 0.05, Table 2).

#### 3.6.2. Hydrophobicity

Strains EF-448, EF-798, and EF-804 showed relatively high hydrophobicity ability and did not differ significantly from different *E. faecalis* strains (*p* > 0.05, Table 3).

### 3.7. Tolerance Analysis of Screened Strains

**Figure 5:**
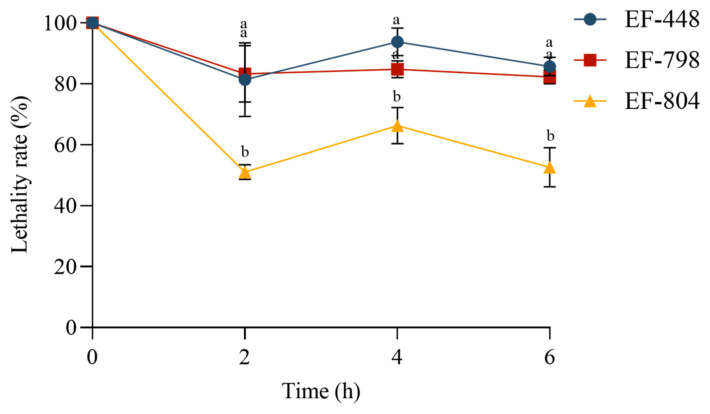
Tolerance analysis of screened strains. Bars with different letters for each mean value indicate statistically significant differences (*p* < 0.05).

The survival of the strains in simulated intestinal fluid decreased with the increase in treatment duration. EF-448 and EF-798 still had high survival at 6 h, with 85.71% and 82.32%, respectively. EF-804 has relatively low survival rates, remaining at 50% at various times compared with others. It was shown that the three isolated strains were generally well tolerated by the simulated intestinal fluid (Figure 5).

### 3.8. Antioxidant Capacity

**Figure 6:**
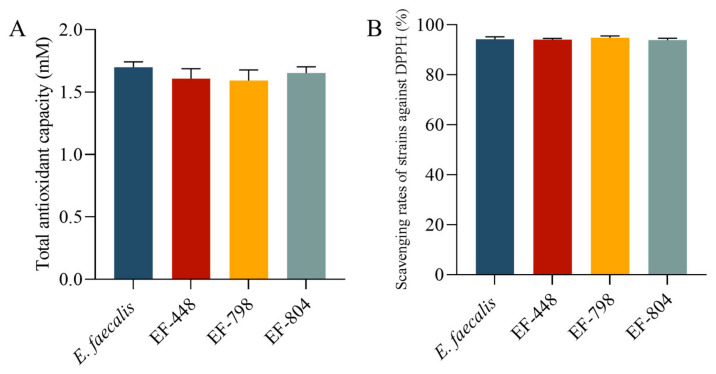
(**A**) The antioxidant capacity and (**B**) scavenging rates of strains against the DPPH of antioxidant capacity of the strain.

There was no significant difference among T-AOC of the strains *E. faecalis*, EF-448, EF-798, and EF-804 in terms of the total antioxidant capacity and DPPH free radical scavenging ability (*p* > 0.05, Figure 6).

### 3.9. Safety Evaluation

#### 3.9.1. Hemolytic Activity

**Figure 7:**
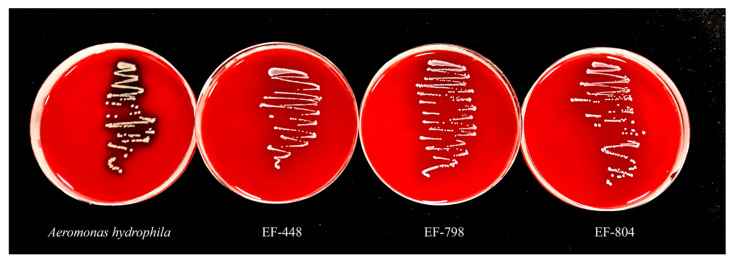
Hemolytic activity.

The results of strain hemolysis showed that none of the three strains, EF-448, EF-798, and EF-804, were hemolytic except for the positive control *Aeromonas hydrophila* which had β hemolysis (Figure 7).

#### 3.9.2. Mutagenic Strains Safety Testing

The results showed that there was no mortality or intoxication in the experimental group in comparison with the control group (Table 4).

## 4. Discussion

The ARTP mutation breeding system has demonstrated the ease of use, high safety, and high genetic stability of mutants in the mutagenesis of various microorganisms using this new method. The effect of the plasma on the microorganisms depends on the operating conditions, such as power input, treatment distance, gas flow rate, and treatment time. The most critical of these is the treatment duration [8]. The lethality of *E. faecalis* increases with its increasing mutagenicity time due to DNA and protein damage caused by the injection of reactive ions into the cell, which generates multiple repair sites during the repair process, resulting in the creation of new mutants [14,24]. Mortality is directly proportional to treatment time. Therefore, determining the optimal time for mutagenesis is critical [25]. The mutations induced by ARTP are random, and there is no direct linear relationship between lethality and mutations. However, it was found that the strains were prone to mutations when the lethality was 85–95% [26,27]. Therefore, the optimal mutation times chosen in this study were 40 s, 50 s, and 60 s.

In recent years, as global fishmeal resources have become scarce and prices have continued to rise, the proportion of plant protein sources in feed has gradually increased. However, high a proportion of vegetable protein sources in feed leads to damage to the intestines and reduced digestive function and is harmful to animal health [28]. The addition of probiotics has been demonstrated to ameliorate intestinal barrier damage by modulating the composition of the gut microbiota [29]. The probiotics screened in this study as feed additives can colonize the digestive tract to increase digestive enzymes and enhance nutrient absorption. As fermentative strains, they can increase the digestibility of plant ingredients and reduce the negative effects of plant ingredients [30]. Carbohydrate metabolism in aquatic organisms is limited. Starch is the primary digestible polysaccharide found in plant feeds used in aquaculture and has a significant effect on the growth of these organisms. The growth process relies heavily on the presence of amylase, an enzyme that plays a vital role in starch digestion [7]. Consequently, enhancing the activity of starch enzymes can markedly augment feed availability by facilitating carbohydrate utilization. Protein is a vital ingredient for the growth, development, and sustenance of aquatic organisms. This feed element is the most expensive component, and its digestibility may be enhanced by augmenting protease activity, hence improving protein usage [31]. The fat is used to replace the protein and provide energy [32]. Therefore, the increase in neutral protease, lipase, and α-amylase activity in the *E. faecalis* contributed to the utilization of the feeding in this study.

Zhang et al. used ARTP to screen the mutant mut80 for a 90.54% and 143.10% increase in the amylase and protease activities of *Bacillus licheniformis* XS-4 [18]. The resequencing results showed that ARTP resulted in effective mutations in the amino acid metabolism genes *ykvZ* and *alsT*. Protease-related synthetic genes (*aprX*) were up-regulated, while the amylase gene (*amyA*) increased 11.26-fold. Zhao et al. screened *Penicillium oxalicum* for a strain with high production of starch-degrading enzymes by four rounds of EMS mutagenesis and two rounds of Co60-γ-ray mutagenesis and genetic engineering techniques as well as up-regulation of amylase-related synthetic genes (*PoxGA15A*, *PoxAmy13A*, *POX_b02418*, and *PoxAmyR*) [33]. ARTP mutagenesis treatment for 180 s increased the acid, neutral, and total protease activities of *Aspergillus oryzae* strain 3.042 by 54.7%, 17.3%, and 8.5%, respectively. However, the alkaline protease activity was reduced by 8.1% compared to the original strain [34]. This aligned with our observations. The three strains were screened with a 180% increase in α-amylase activity, a 30% increase in lipase, and a 40% increase in neutral protease. The reactive species in the helium RF APGD plasma jets could also cause changes in the molecular structure of lipase, leading to an increase in enzyme activity [35]. These results indicate that ARTP technology has emerged as a potent method for increasing enzyme synthesis.

The genetic stability of the mutant strain is crucial for industrial applications, since it indicates all potential changes at the genetic level. In the present study, the genetic stability of the mutant strain was investigated using continuous subcultures [36]. Even after nine rounds of fermentation, the digestive enzyme yields of EF-448, EF-798, and EF-804 remained high, indicating that the three mutant strains have good genetic stability. These findings were comparable to those of Zhang et al., who examined the genetic stability of the ARTP mutant by culturing the strain for six generations. Their results demonstrated that the chitosanase activity remained relatively constant throughout this period [11]. This may be due to the repair action of SOS, which is a highly error-tolerant process. Various mismatch sites are formed and stabilized during the repair process, resulting in genetic characteristics and mutants [37]. As a result, it is demonstrated that the ARTP mutation is a promising technique for creating extremely genetically stable mutants.

Adherence to intestinal epithelial cells is favorably correlated with probiotic auto-aggregation and hydrophobicity [38]. Consequently, probiotics with greater auto-aggregation and hydrophobicity have a greater ability to adhere to the cells lining the gut, thereby improving gut health [20].

In this study, all strains screened were found to have high auto-aggregation capacity, and the auto-aggregation capacity after 24 h of incubation exceeded that after 2 and 4 h of incubation. which was similar to the results of Angmo et al. [20]. Furthermore, the auto-aggregation capacity exhibited a positive correlation with the duration of the experiment. The auto-aggregation capacity was enhanced by the rise in the aggregation-promoting component during incubation [39].

Cell surface hydrophobicity is a various characteristic between different strains and is influenced by the glycoprotein molecules in the surface of microbial cells. As a result, noticeable variations in hydrophobicity can be found even among individuals of the same species [40]. At least 40% hydrophobicity is an essential prerequisite for probiotics [41]. EF-448, EF-798, and EF-804 had high cell surface hydrophobicity (90%) in this study. Three mutant isolates exhibited a ropy phenotype on MRS solid medium. After high-speed centrifugation of the liquid MRS medium, the supernatant still contained mucilaginous substances, presumed to be capsular polysaccharides (CPS). This substance is intimately associated with the cell and has a direct influence on the cell’s ability to perform auto-aggregation as well as its hydrophobicity [42,43]. There is a strong correlation between cell surface hydrophobicity and auto-aggregation, and hydrophobicity flanks the adhesive effect of the strain on the gut [42].

Probiotics can adhere to the intestinal mucosa and mucus, produce antimicrobial substances to fight against pathogens, and interact with gut-associated lymphoid tissue (GALT) to inactivate harmful components of the intestinal contents [44]. In addition to adherence, the capacity to endure throughout the digestive system is also essential. The three strains tested in this study were well tolerated in simulated intestinal fluid (pH = 7.8).

Oxidative stress occurs when there is an imbalance between the formation of free radicals and the ability of cells to eliminate them, and oxidative stress is a key factor in the metabolic and physiological changes in organisms and in various diseases [45]. The antioxidant activity of probiotics may help to protect against oxidative damage by reducing free radicals and thus the oxidative stress response [46]. Glutathione is an antioxidant compound that can participate in the antioxidant mechanism of the intestinal mucosa and protect it from oxidation-induced tissue damage [47]. Yang et al. identified functional genes related to glutathione synthesis (gshAB) in the genome of mutant strains [42].

Lou et al. demonstrated that *E. faecalis* exhibited a significant ability to scavenge DPPH free radicals (97%), which aligns with our findings [48]. This suggested that the three mutant isolates have the potential to be used as antioxidant strains in functional food and feed. Although it was found that the total antioxidant capacity and DPPH free radical scavenging ability of the strains were not altered after ARTP mutagenesis in the present study. Newly acquired probiotics need to be shown to be non-pathogenic over a period of time to determine their safety before they can be used. Jin et al. studied the hemolytic activity of 15 strains of *Lactobacillus*. All tested isolates were shown to be negative for a hemolytic reaction when grown in Columbia sheep blood agar [21]. This is consistent with our findings that all isolated strains had nonhemolytic activity. Hemolysis is usually associated with bacterial pathogenicity, but there is no universal correlation. Genetic analyses of virulence and pathogenicity are also required for a comprehensive assessment of strain pathogenicity. Future study should also focus on analysis safety at the genomic level and establishing its transcriptome, proteome, or metabolome for a fine selection of safe probiotic strains [49]. In this work, a probiotic was injected into *M. rosenbergii*, and no mortality was observed for 14 days. There were no safety concerns in either in vivo or in vitro safety studies of EF-448, EF-798, and EF-804. This has suggested that the mutagenic strains can be considered safe and suitable for use in production practices, allowing further evaluation of the potential role of the strains in vivo.

## 5. Conclusions

Ultimately, we utilized ARTP mutagenesis to acquire three genetically stable and exceptionally efficiency mutant strains, namely EF-448, EF-798, and EF-804. These strains exhibited a remarkable 180% increase in amylase activity, a 30% increase in lipase activity, and a 40% rise in neutral protease activity, respectively. Importantly, the mutant strain retains the superior hydrophobicity, auto-aggregation capacity, and comparative antioxidant capacity of the original strain. Furthermore, it exhibits a higher level of digestive enzyme production compared to the original strain.

## Figures and Tables

**Table 1:** Re-screening of potential probiotic enzyme production activity.

Strains Number	α-Amylase(U/dL)	Neutral Protease (nmol/min/mL)	Lipase (U/mL)	Overall Score (%)
EF-513	43.07 ± 2.98	20.16 ± 0.85	0.53 ± 0.01	91.94 ± 0.99
EF-804	43.03 ± 0.63	20.40 ± 0.43	0.50 ± 0.04	91.37 ± 1.50
EF-494	40.68 ± 2.12	21.16 ± 1.10	0.45 ± 0.01	89.01 ± 3.98
EF-798	40.50 ± 0.39	20.40 ± 0.35	0.46 ± 0.04	88.51 ± 1.41
EF-783	49.27 ± 1.46	16.41 ± 0.29	0.43 ± 0.03	86.22 ± 1.62
EF-448	44.66 ± 2.81	15.65 ± 1.26	0.50 ± 0.01	83.53 ± 4.64
EF-812	45.56 ± 1.81	14.49 ± 0.80	0.48 ± 0.08	81.51 ± 2.65
EF-536	47.07 ± 0.24	14.89 ± 0.82	0.43 ± 0.02	81.44 ± 1.97
EF-782	49.45 ± 1.35	13.19 ± 0.13	0.46 ± 0.01	81.34 ± 1.16
EF-816	49.63 ± 3.74	11.67 ± 0.16	0.51 ± 0.03	80.52 ± 3.33
EF-534	43.75 ± 0.63	17.28 ± 0.88	0.35 ± 0.06	80.46 ± 0.86
EF-811	43.39 ± 0.56	12.55 ± 0.28	0.54 ± 0.01	78.22 ± 1.16
EF-502	40.50 ± 0.94	13.04 ± 1.26	0.54 ± 0.00	76.74 ± 2.51
EF-807	44.29 ± 0.45	12.17 ± 0.33	0.47 ± 0.01	75.77 ± 1.26
EF-454	41.22 ± 0.00	12.95 ± 0.21	0.49 ± 0.01	75.32 ± 0.76
EF-775	41.31 ± 1.86	13.52 ± 0.43	0.43 ± 0.05	74.36 ± 3.01
EF-795	44.48 ± 1.03	12.97 ± 1.19	0.43 ± 0.03	74.12 ± 3.44
EF-452	47.32 ± 1.02	9.57 ± 0.50	0.48 ± 0.01	73.51 ± 1.15
EF-808	44.20 ± 0.41	11.16 ± 0.67	0.45 ± 0.00	72.94 ± 0.53
EF-531	41.76 ± 0.31	12.83 ± 0.13	0.41 ± 0.01	72.64 ± 0.32
EF-453	40.68 ± 1.57	11.81 ± 0.15	0.47 ± 0.01	72.10 ± 1.45
EF-778	42.85 ± 0.68	9.75 ± 0.92	0.51 ± 0.00	71.36 ± 1.06
EF-800	44.20 ± 1.03	9.38 ± 2.27	0.48 ± 0.07	70.88 ± 2.61
EF-451	43.12 ± 3.97	10.36 ± 0.36	0.45 ± 0.05	70.71 ± 3.24
EF-450	41.22 ± 2.59	8.70 ± 0.11	0.53 ± 0.02	68.78 ± 2.23
EF-449	41.85 ± 2.91	8.55 ± 0.96	0.52 ± 0.01	68.66 ± 1.80
EF-781	41.76 ± 0.68	9.24 ± 1.44	0.48 ± 0.01	68.55 ± 2.53
EF-828	44.84 ± 1.19	8.15 ± 0.25	0.44 ± 0.07	67.58 ± 2.20
EF-505	43.75 ± 0.09	6.01 ± 0.96	0.55 ± 0.01	66.61 ± 1.49
EF-788	43.84 ± 1.20	6.34 ± 0.40	0.49 ± 0.02	65.19 ± 0.83
EF-791	40.41 ± 0.54	8.21 ± 0.35	0.47 ± 0.02	65.09 ± 0.34
EF-797	41.40 ± 0.86	8.37 ± 0.25	0.45 ± 0.08	64.70 ± 2.41
EF-799	41.40 ± 1.83	6.41 ± 0.75	0.47 ± 0.01	64.63 ± 0.95
EF-792	40.41 ± 0.54	6.52 ± 0.23	0.52 ± 0.04	64.04 ± 1.29
EF-784	46.19 ± 2.67	4.24 ± 0.06	0.50 ± 0.01	63.33 ± 1.99
EF-785	40.23 ± 1.11	6.23 ± 0.45	0.53 ± 0.01	63.19 ± 0.56
EF-516	40.41 ± 0.72	7.07 ± 0.06	0.47 ± 0.02	62.87 ± 0.08
EF-805	40.59 ± 1.19	13.84 ± 0.49	0.07 ± 0.01	61.46 ± 1.43
EF-564	40.59 ± 1.40	7.39 ± 1.07	0.40 ± 0.01	61.24 ± 2.50
EF-532	41.76 ± 0.47	6.90 ± 0.28	0.40 ± 0.00	60.99 ± 0.79
EF-375	40.05 ± 1.27	6.52 ± 0.87	0.45 ± 0.01	60.78 ± 2.16
EF-771	41.31 ± 1.67	4.24 ± 0.23	0.52 ± 0.00	59.96 ± 1.09
EF-780	42.76 ± 0.86	4.38 ± 0.49	0.45 ± 0.07	59.01 ± 2.41
EF-383	40.41 ± 0.72	7.32 ± 0.19	0.35 ± 0.03	58.93 ± 1.78
EF-794	40.50 ± 0.63	3.26 ± 0.06	0.44 ± 0.02	57.86 ± 0.74
EF-786	48.09 ± 1.01	-	0.50 ± 0.05	56.95 ± 1.43
EF-527	41.67 ± 0.24	3.75 ± 0.72	0.45 ± 0.01	56.94 ± 1.79
EF-603	42.40 ± 2.25	4.75 ± 0.38	0.38 ± 0.02	56.88 ± 1.30
EF-535	42.58 ± 0.41	3.70 ± 0.63	0.42 ± 0.02	56.66 ± 2.01
EF-819	43.84 ± 1.27	0.76 ± 0.06	0.52 ± 0.01	55.59 ± 1.30
EF-815	45.29 ± 1.59	-	0.52 ± 0.02	55.32 ± 1.81
EF-263	40.32 ± 0.33	4.28 ± 1.13	0.40 ± 0.03	55.09 ± 3.08
EF-774	40.23 ± 1.89	2.17 ± 0.50	0.50 ± 0.00	54.63 ± 2.46
EF-824	46.46 ± 2.43	-	0.47 ± 0.03	54.37 ± 1.88
EF-533	40.95 ± 1.65	3.32 ± 0.28	0.41 ± 0.01	54.14 ± 1.35
EF-522	41.40 ± 0.59	2.12 ± 0.09	0.46 ± 0.01	54.00 ± 0.17
EF-820	44.75 ± 0.72	-	0.49 ± 0.02	53.62 ± 0.24
EF-823	43.93 ± 1.24	-	0.50 ± 0.01	53.39 ± 1.05
EF-525	43.93 ± 1.03	-	0.49 ± 0.01	53.25 ± 1.20
EF-790	43.75 ± 1.46	-	0.49 ± 0.04	52.96 ± 0.86
EF-509	42.94 ± 0.86	-	0.50 ± 0.02	52.58 ± 0.27
EF-793	40.95 ± 0.16	2.88 ± 0.09	0.40 ± 0.04	52.53 ± 0.93
EF-827	45.02 ± 0.41	-	0.45 ± 0.04	52.44 ± 1.60
EF-779	42.12 ± 1.42	-	0.51 ± 0.01	52.35 ± 1.38
EF-810	43.66 ± 1.22	-	0.47 ± 0.06	52.32 ± 3.13
EF-395	41.67 ± 1.04	-	0.52 ± 0.03	52.27 ± 1.43
EF-832	44.20 ± 1.18	-	0.44 ± 0.01	51.64 ± 1.20
EF-501	42.40 ± 2.09	-	0.48 ± 0.03	51.59 ± 0.80
EF-796	42.22 ± 0.63	-	0.49 ± 0.02	51.14 ± 0.75
EF-529	43.03 ± 1.84	-	0.44 ± 0.01	50.62 ± 1.33
EF-530	43.12 ± 1.24	-	0.44 ± 0.01	50.62 ± 1.13
EF-514	41.67 ± 1.42	0.87 ± 0.44	0.42 ± 0.05	50.53 ± 0.17
EF (control)	15.82 ± 1.81	13.12 ± 0.74	0.34 ± 0.05	49.82 ± 3.01
EF-537	43.03 ± 1.19	-	0.41 ± 0.00	49.50 ± 0.68
EF-528	41.22 ± 0.98	-	0.45 ± 0.01	49.44 ± 0.64
EF-526	41.85 ± 0.77	-	0.43 ± 0.06	49.39 ± 1.89
EF-366	42.94 ± 2.11	-	0.41 ± 0.02	49.35 ± 1.85
EF-607	41.13 ± 1.82	2.07 ± 0.57	0.33 ± 0.03	49.12 ± 2.96
EF-523	40.68 ± 0.16	-	0.45 ± 0.02	49.08 ± 0.56
EF-770	40.77 ± 0.39	-	0.44 ± 0.04	48.80 ± 1.50
EF-517	41.13 ± 0.94	-	0.43 ± 0.02	48.67 ± 1.12
EF-226	40.77 ± 5.65	-	0.43 ± 0.02	48.24 ± 5.00
EF-611	42.22 ± 1.01	-	0.34 ± 0.02	46.24 ± 1.06
EF-379	40.59 ± 1.89	-	0.36 ± 0.03	45.70 ± 0.96

Note: Overall score (%) = (α-amylase activity/maximum α-amylase activity) × 100 × 0.4 + (neutral protease activity/maximum neutral protease activity) × 100 × 0.4 + (lipase activity/maximum lipase activity) × 100 × 0.2. “-” means no detection.

**Table 2:** Auto-aggregation of different strains.

Strain	Auto-Aggregation (%)		
	2 h	4 h	24 h
EF	10.17 ± 0.72	14.27 ± 2.33	94.01 ± 0.72
EF-448	9.18 ± 1.05	11.56 ± 1.01	93.05 ± 0.82
EF-798	8.89 ± 0.66	13.77 ± 1.57	92.67 ± 0.47
EF-804	9.78 ± 0.57	12.07 ± 0.44	91.03 ± 1.05

**Table 3:** Hydrophobicity of different strains.

Strain	Hydrophobicity (%)
EF	93.98 ± 0.41
EF-448	93.86 ± 0.33
EF-798	94.59 ± 0.04
EF-804	94.42 ± 0.07

**Table 4:** Mutagenic strains EF-494, EF-798 and EF-804 safety testing.

Strain	Concentration (CFU/mL)	Death Rate (%)
Sterile saline	-	0
EF-448	10^8^	0
	10^10^	0
EF-798	10^8^	0
	10^10^	0
EF-804	10^8^	0
	10^10^	0

## Data Availability

The original contributions presented in the study are included in the article, further inquiries can be directed to the corresponding authors.
